# Genetic Diversity of Cameroon Cattle and a Putative Genomic Map for Resistance to Bovine Tuberculosis

**DOI:** 10.3389/fgene.2020.550215

**Published:** 2020-11-17

**Authors:** Rebecca Callaby, Robert Kelly, Stella Mazeri, Franklyn Egbe, Lindert Benedictus, Emily Clark, Andrea Doeschl-Wilson, Barend Bronsvoort, Mazdak Salavati, Adrian Muwonge

**Affiliations:** ^1^Centre for Tropical Livestock Genetics and Health, The Royal (Dick) School of Veterinary Studies, The Roslin Institute, University of Edinburgh, Edinburgh, United Kingdom; ^2^The Royal (Dick) School of Veterinary Studies, The Roslin Institute, University of Edinburgh, Edinburgh, United Kingdom; ^3^School of Life Sciences, University of Lincoln, Lincoln, United Kingdom

**Keywords:** susceptibility, admixture, *Bos indicus*, Cameroon, bovine tuberculosis

## Abstract

Bovine Tuberculosis (bTB) caused by *Mycobacterium bovis* is a livestock disease of global economic and public health importance. There are currently no effective vaccines available for livestock and so control relies on animal level surveillance and pasteurization of dairy products. A new alternative control approach is to exploit the genetic variability of the host; recent studies have demonstrated that breeding European taurine cattle, such as Holsteins for increased resistance to bTB is feasible. The utility of such an approach is still unknown for African cattle populations. This study aims to assess genetic variation in bTB resistance and the underlying genomic architecture of cattle in Cameroon. We conducted a cross-sectional study of 2,346 slaughter cattle in Cameroon. Retropharyngeal lymph node samples were collected and cultured on Lowenstein Jensen media and the BACTEC MGIT 960 system, and *M. bovis* was identified using the Hain® Genotype kits. A total of 153 cattle were positive for *M. bovis* and were archived along with a random selection of negative samples. In this study, we genotyped archived samples from 212 cattle. Their genomic diversity was characterized using PCA, hierarchical clustering and admixture analysis. We assessed genetic variation in bTB resistance using heritability analysis and compared quantitative trait loci. Previous research on this study population have shown that Fulani cattle are more susceptible to bTB than mixed breeds. However, here we show that these apparent phenotypic differences in breeds are not reflected by clear genomic differences. At the genetic level, both the Fulani and mixed cattle show similar patterns of admixture with evidence of both taurine and indicine ancestry. There was little European taurine introgression within the studied population. Hierarchical clustering showed clusters of cattle that differed in their susceptibility to bTB. Our findings allude to bTB resistance being polygenic in nature. This study highlights the potential for genetic control of bTB in Africa and the need for further research into the genetics of bTB resistance within African cattle populations.

## 1. Introduction

Bovine tuberculosis (bTB) caused by *Mycobacterium bovis* is a major zoonotic livestock disease causing a chronic respiratory condition characterized by weight loss and poor welfare and eventually death. In low-and middle-income countries (LMICs) it is estimated that *M. bovis* is responsible for ~1.4% of human TB cases, which equates to an estimated 70,000 new human infections annually in Africa (Olea-Popelka et al., 2017). *Mycobacterium bovis* is a member of the *Mycobacterium tuberculosis* complex, it primarily infects humans via consumption of unpasteurized milk, poorly cooked meat and close contact with infected animals. Unfortunately, the risks of bTB infection from milk are poorly understood by the livestock keepers in Cameroon, who are mainly pastoralist (Ayele et al., 2004; Kelly et al., 2016). There are currently no effective vaccines available for use in livestock and public health control relies on pasteurization of dairy products, surveillance at post-mortem examination in slaughterhouses and active surveillance of cattle as part of test and slaughter programmes. LMICs in sub-Saharan Africa currently employ passive abattoir surveillance through official veterinary services, while pasteurization is carried out at the household level in many settings where there is no centralized collection of milk. In these settings, bTB is considered endemic, with the prevalence in cattle estimated to range from 6 to 20% depending on the region and diagnostic tools used (Asante-Poku et al., 2014; Dibaba and Daborn, 2019).

*M. bovis* has a wide host range, which includes domestic and wild bovids, small ruminants, swine and cervids; which means that control requires a strong coordinated multi-disciplinary, trans-boundary approach. Unfortunately, most LMICs have poorly resourced veterinary services and do not have the means to implement bTB control strategies, instead they rely on controlling the risk of exposure through abattoir surveillance. At the same time LMICs, encouraged and funded by many charities, have widely adopted dairy development strategies with the aim of aiding livelihood and community development (Tambi, 1991; Bill & Melinda Gates Foundation, 2012; Heifer International, 2019). Although this brings clear nutritional and economic benefits to those keeping dairy cattle, there are concerns that the current strategy based on the introduction of exotic (European taurine) genetic characteristics, i.e., improved milk production and faster growth in the local breeds of Africa, may also result in increased susceptibility to and risk of several zoonotic diseases including bTB (Hanotte et al., 2000; Coffie et al., 2015; Bahbahani et al., 2018; Opoola, 2018; Opoola et al., 2019).

There is epidemiological evidence from Ethiopia that there are breed differences in susceptibility to *M. bovis* with *Bos indicus* breeds appearing to be less susceptible compared to European *Bos taurus* breeds (Vordermeier et al., 2012). Breed introductions are usually based on a centripetal model, i.e., governments import taurine breeds into centrally located breeding facilities, which then distribute “improved breeds” outwards into rural areas (Mekonnen et al., 2019). In Ethiopia, for example, concerns have been raised that this approach could inadvertently spread bTB from urban areas where the rates are high to rural settings where they are lower (Mekonnen et al., 2019).

To mitigate such risks new approaches to add to the current bTB control tool kit are urgently required. One such approach is breeding for resistance to bTB. This is based on exploiting the observed genetic variation in resistance to *M. bovis* infection in cattle (Brotherstone et al., 2010; Bermingham et al., 2014; Tsairidou et al., 2014). Furthermore, recent research demonstrated that breeding for increased resistance to bTB is feasible, and has generated the necessary tool set to carry out selective breeding for bTB resistance (Banos et al., 2017; Tsairidou et al., 2018). The UK dairy industry has recently implemented pedigree selection into the “TB Advantage” genetic evaluation index that enables breeders and farmers to select bulls with greater bTB resistance (Banos et al., 2017). This has proven to be popular with farmers and the breeding industry.

Prevalence of bTB has been increasing in Cameroon since 2003 (Awah-Ndukum et al., 2012; Egbe et al., 2016). There is, therefore, an opportunity to help prevent or at least reduce the scale of an emerging bTB epidemic in African dairy cattle through genetic selection. For this approach to gain traction, we need to reconcile the potential disparity between phenotypic and genotypic characteristics of cattle in Africa. Our previously published results showed that even after controlling for other factors, breed is an important factor in explaining the increased risk of infection, with the Fulani breed appearing to be more susceptible to bTB than the mixed breed group (Kelly et al., 2018).

In this paper we investigate this possibility by using genetic and phenotypic data from archived samples taken from cattle diagnosed as bTB positive and negative during a cross-sectional study of bTB in Cameroon (Egbe et al., 2016, 2017; Kelly et al., 2018) to assess genetic variation in bTB resistance and the underlying genomic architecture of cattle breeds in Cameroon.

## 2. Materials and Methods

### 2.1. Cross-Sectional Study of bTB in Cameroon Abattoirs

**Figure 1:**
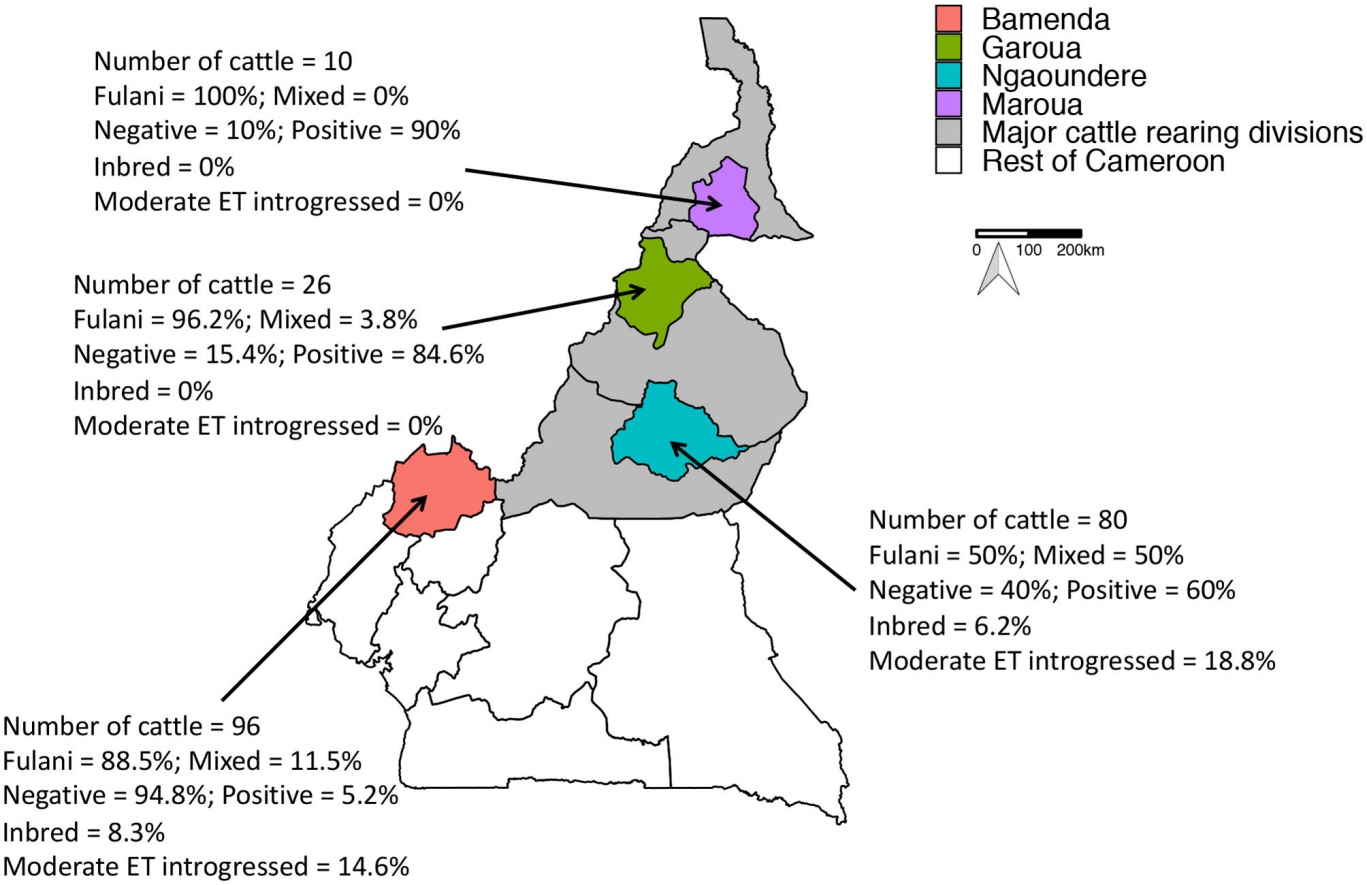
Map of Cameroon showing the major cattle rearing divisions and the divisions where the abattoirs are located. The number of genotyped cattle per abattoir, the proportion of mixed and Fulani cattle, proportion of *M. bovis* positive cattle, proportion of inbred cattle and proportion moderately European taurine introgressed cattle per abattoir is also shown.

Between 2012 and 2013, a cross-sectional study of 2,346 slaughter cattle was conducted in four regions of Cameroon (see Figure 1). As animals came into each abattoir the age, as estimated by the dentition score (individuals were defined as young if they have a dentition score between 0 and 2, i.e., no permanent incisors; old individuals have a dentition score between 3 and 5), sex and breed as defined by the local abattoir employees (mixed breed or Fulani) was recorded. A heparinized blood sample was collected and local Ministry of Livestock, Fisheries and Industrial Agriculture (MINEPIA) inspectors carried out a post-mortem on the carcass, looking for evidence of granulomatous bTB-like lesions. If an animal had bTB-like lesions then up to 3 lesions per animal were collected for culture and a random sample of retropharyngeal lymph nodes from non-lesioned animals was also collected for comparison. More in depth detail about the study design and diagnostic tests in Egbe et al. (2016).

Briefly, inoculum was prepared from lymph nodes and a portion cultured in Mycobacterial Growth Indicator Tubes (MGIT), these were incubated for 8 weeks on the BACTEC MGIT 960 automated culture system (Becton, Dickinson and Company, 1 Becton Drive, Franklin Lakes, NJ, USA) following the manufacturer's instructions (Egbe et al., 2016). Negative MGITs were examined visually after 56 days for growth before discarded (Egbe et al., 2016). Another portion of the inoculum was cultured on Lowenstein Jensen (LJ) media (one supplemented with pyruvate and the other with glycerol) and observed weekly for up to 12 weeks. After 12 weeks, if no growth was observed then they were classified as culture negative (Egbe et al., 2016). If any growth was suspected, on both media, a smear was prepared, stained according to the Ziehl-Neelsen (ZN) method and examined with a microscope (100× magnification) to assess the presence of acid-fast bacilli (Lumb et al., 2013). All samples with observed acid-fast bacilli were screened for the presence of *M. bovis* using the Hain GenoType® MTBC assay (Hain Lifescience®, GmbH, Nehren, Germany) (Egbe et al., 2016).

*M. bovis* cases were defined as animals that have one or more lesions which were positive by one or more culture methods and were confirmed by typing using the Hain GenoType® MTBC assay. Whilst, controls are animals which had a *M. bovis* negative culture of the retropharyngeal lymph node.

### 2.2. Sample Selection

Out of the 2,346 animals in the cross-sectional study, 153 animals were cultured and confirmed positive for *M. bovis*. Due to logistical and storage limitations in Cameroon, samples from 227 animals where archived. This consisted of 97 *M. bovis* positive animals and a random selection of 130 control animals. The random selection took into account the age and breed structure in each of the abattoirs and so it is representative of the original study. In 2019, all the archived samples were genotyped and formed the basis for this paper. Following genotyping quality control checks (described below), 84 *M. bovis* culture positive and 128 *M. bovis* culture negative animals were included in the analyses.

### 2.3. Genotyping and Quality Control

DNA was extracted from the frozen archived bovine lymph nodes using the Qiagen DNeasy® Blood & Tissue Kit. The “Purification of Total DNA from Animal Tissues (Spin-Column Protocol)” was used following the manufacturer's instructions.

Frozen lymph node samples stored at −80°C were brought to room temperature and ~25 mg was sliced into tiny pieces on a sterile petri dish using sterile disposable surgical blades. The sliced tissues were transferred to a 1.5 ml screw cap microtube and heat-treated for 10 min at 95°C in a water bath. Tubes were retrieved from the water bath and 180 μl of Buffer ATL and 20 μl of proteinase K (provided with the kit) was added. Tubes were vigorously vortexed to mix and the tissues were completely lysed by incubating at 56°C for 3 h in a heater block. The rest of the procedure was done strictly following the manufacturer's instructions. Purified DNA was first eluted from the spin columns with 60 μl of the elution buffer to increase the DNA concentration. To maximize the DNA yield, a second elution with 180 μl of elution buffer was done. The concentration of the purified DNA were assessed using the Qubit.

The cattle were genotyped using the Illumina BovineHD 777K BeadChip, which included 777,962 SNPs, of which 735,293 were autosomal (Illumina, 2015). In the archived subset there were lymph node tissue samples from 172 Fulani cattle and 56 mixed breeds. In order to assess the genomic architecture, Illumina BovineHD 777K BeadChip array datasets where obtained from an additional 266 reference animals (Bovine HapMap Consortium, 2009; Bahbahani et al., 2017). These reference animals represent European taurine breeds (Holstein *n* = 63 and Jersey *n* = 36), African taurine breeds (N'Dama *n* = 24, Muturu = 10), Asian zebu *Bos indicus* breeds (Nelore *n* = 35, Gir = 30), and West African admixed breeds (Bunaji *n* = 23, Friesian-Bunaji = 24, Sokoto Gudali = 21).

Quality control checks was carried out using the program PLINK v1.90 (Chang et al., 2015; Purcell and Chang, 2018). SNPs with a minor allele frequency (MAF) of <0.01 or a call rate of <90% were removed. No Hardy-Weinberg equilibrium cut-off was used to avoid removal of informative SNPs. Individuals that were related with a degree of relatedness >2 according to the KING relatedness algorithm were removed (Manichaikul et al., 2010). This left a total of 500,929 autosomal SNPs and 356 cattle, of which 212 animals were from Cameroon.

### 2.4. Genetic Diversity

Investigation of the genetic diversity was carried out by comparing the Fulani and the mixed breed animals from Cameroon (*n* = 212) to 144 reference animals that passed quality control checks (Supplementary Table 1).

#### 2.4.1. Population Genetic Structure

Principal components analysis (PCA) was performed using the *pca* function of PLINK v1.90 (Chang et al., 2015; Purcell and Chang, 2018) to provide an insight into the population structure of the cattle breeds.

Next, hierarchical clustering was performed on the genome-wide identity-by-state (IBS) pairwise distances between individuals using the *SNPRelate* package in R version 3.5.0 (Zheng et al., 2012; R Core Team, 2018). Subgroups of individuals were determined using a Z-score threshold of 15 based upon individual dissimilarities to define groups of individuals in the hierarchical cluster analysis. An outlier threshold of 5 was also set, this means that groups with ≤ 5 animals are considered outliers. For comparison the dendrogram was redrawn using breed and population type to determine the groups.

Population genetic structure was also evaluated using the ADMIXTURE software tool (Alexander et al., 2009) to determine the European taurine, Asian zebu *Bos indicus* and African taurine ancestries at the genome-wide level. Variants in high linkage disequilibrium (LD) with each other were removed prior to analysis. The LD pruning criteria applied was to remove any SNP that had an r-squared >0.2 with another SNP within a 200-SNP window; for a sliding window of 10 SNPs at a time, which resulted in 55,361 markers (out of 500,929 markers) left for analysis. A 5-step expectation–maximization (EM) algorithm was used. In addition, 10-fold cross validation was performed with 200 bootstrap resampling runs to estimate the standard errors for each cluster level (*K* = 2–12). The output was plotted using the *pophelper* package for R (Francis, 2017). The optimal number of clusters was determined from the cross-validation plot.

To provide further insights into the Fulani and Mixed compositions related to the other groups, the ADMIXTURE analysis was also run in a supervised mode, with European taurine, African taurine, and Asian zebu (*Bos indicus*) and Admixed groups pre-specified.

#### 2.4.2. Genetic Differentiation and Inbreeding Coefficients

To estimate the degree of genetic differentiation in the cattle populations, fixation indices (Fst) were calculated using the Weir and Hill (2002) relative beta estimator method as implemented by the *snpgdsFst* function in the *SNPRelate* package (Weir and Hill, 2002; Zheng et al., 2012; Buckleton et al., 2016). In addition, the *het* function of PLINK v1.90 (Purcell and Chang, 2018) was used to calculate the inbreeding coefficient estimate, F, for each individual.

#### 2.4.3. Ascertainment Bias of the SNP Array

Differences in the inbreeding coefficient may also be due to the ascertainment bias of the SNP array. To assess this ascertainment bias, linkage disequilibrium, *r*^2^ and D′, was calculated between pairs of SNPs within a 50 SNP window using PLINK v1.90 (Purcell and Chang, 2018) for each breed. The *r*^2^ was then plotted against the window size (bp distance between first and last SNP within the window) and a second degree polynomial fitted to show the trend in *r*^2^ for each breed. The average *r*^2^ and D′ also calculated for each breed.

### 2.5. Genetics of bTB Resistance

#### 2.5.1. Association Between bTB Infection Status and Breed

Binomial generalized linear models with logit link functions were used to examine the association between breed and bTB infection status in R (R Core Team, 2018). Two separate models were run, the first used the local abattoir employees record of breed to test the association between breed and bTB. Age and sex were included in both models as fixed effects. The second model had the same structure but breed was replaced with the hierarchical clustering definition of subgroups of animals based on genomic information. Abattoir was not included as a fixed effect as not all abattoirs had mixed cattle. This could have large effects on the estimates, as abattoir may absorb all other potential systematic environmental effects on bTB status.

#### 2.5.2. Association With Inbreeding and European Taurine Introgression

European taurine (ET) introgression status was based on the proportion membership to the K value cluster representing European taurine breeds in the admixture analysis (Cluster 3 from *K* = 3). We used the cut-offs from (Mbole-Kariuki et al., 2014) to define “moderate” ET introgressed cattle as individuals with between 1.56 and 12.5% ET introgression (*n* = 29) and cattle with ≤ 1.56% ET background represented the “non-European” introgressed group (*n* = 183). There were no “substantially” ET introgressed cattle with >12.5% ET introgression in this population.

Cattle were defined as inbred using a method adapted from Murray et al. (2013), which we describe here. Non-ET introgressed cattle were categorized as inbred if they had an inbreeding coefficient value of >0.182 (more than 0.10 above the mean for this group). As in Murray et al. (2013), for ET introgressed animals, the best fit linear regression of inbreeding against introgression was found and cattle were excluded if they had an inbreeding coefficient >0.01 above their expected value. This resulted in the identification of 13 inbred cattle, 8 of which showed moderate ET introgression.

We examined the relationship between bTB and (i) ET introgression and (ii) the inbreeding coefficient using binomial generalized linear models with logit link functions in R (R Core Team, 2018). A separate model was built for each. Age and sex were included in the models as categorical fixed effects. Again, abattoir was not included as a fixed effect as some abattoirs did not have any inbred or ET introgressed cattle.

#### 2.5.3. Heritability Analysis

The heritability of bTB resistance in the Cameroon dataset was assessed using linear animal models (Lynch and Walsh, 1998), which are a form of mixed model with fixed and random effects, that can break phenotypic variation down into the different components using the following structure:

$$\begin{array}{l} y=Xb+Za+e \end{array}$$

where *y* is the phenotype of interest (in this case, *M. bovis* status) and *b* is a vector of fixed effects (age, sex, and breed). The random effects, which determine the variance of the trait are the additive genetic (*a*) and residual effects (*e*). *X* and *Z* are all design matrices assigning individuals to their corresponding fixed and random effects. Both random effects were assumed to follow a normal distributions, a N(0,$\sigma_{a}^{2}$G), where $\sigma_{a}^{2}$ is the additive genetic variance and G is the genomic relationship matrix constructed from the inverse of the IBS matrix which was created using the R package *SNPRelate* (Zheng et al., 2012) as described above; the residual error e N(0, $\sigma_{e}^{2}$I) with residual variance $\sigma_{e}^{2}$ and identity matrix I.

The narrow-sense heritability of a trait (*h*^2^) is defined as the proportion of phenotypic variance explained by the additive genetic variance, $h_{2}=\sigma_{a}^{2}/\left(\sigma_{a}^{2}+\sigma_{e}^{2}\right)$. It describes the extent to which differences between individuals are determined by additive genetic effects (Falconer et al., 1996). The heritability analyses were carried out in ASReml version 3.0 (Gilmour et al., 2009).

#### 2.5.4. Genome Wide Association Study

A genome wide association study (GWAS) was carried out on the quality controlled Cameroon dataset using PLINK version 1.90 (Chang et al., 2015; Purcell and Chang, 2018). Linear regression models were run to evaluate the association between bTB and each SNP. Age, sex, and breed, and the first three principal components were included in the models as fixed effects to adjust for population structure. A suggestive threshold (*P* < 1 × 10^−5^ was used in addition to a Bonferroni corrected genome-wide significance threshold of *P* = −*log*_10_(0.05/*n*) = 7.00 (where *n* is the number of SNPs, *n* = 500,929). Regions surrounding 100 Kbp of SNPs which had been identified as being associated with bTB resistance in cattle in the literature (Driscoll et al., 2011; Finlay et al., 2012; Amos et al., 2013; le Roex et al., 2013; Bermingham et al., 2014; Richardson et al., 2016; Raphaka et al., 2017; Tsairidou et al., 2018) were also evaluated with a lower significance threshold of *p* = −*log*_10_(0.05/45780) = 5.96.

## 3. Results

### 3.1. Descriptive Results

A total of 212 animals from Cameroon passed quality control checks, of which 160 animals (75.5%) were Fulani and 52 animals (24.5%) were mixed breed. 84 animals (39.6%) were positive for *M. bovis*. Within the Fulani group 38.8% of animals (62 individuals) were positive for *M. bovis* compared to 42.3% of mixed breed animals (22 individuals). The proportion of individuals positive for *M. bovis* by age, gender and ET introgression and inbreeding status by abattoir is shown in Figure 1.

### 3.2. Genetic Diversity

#### 3.2.1. Population Structure

**Figure 2:**
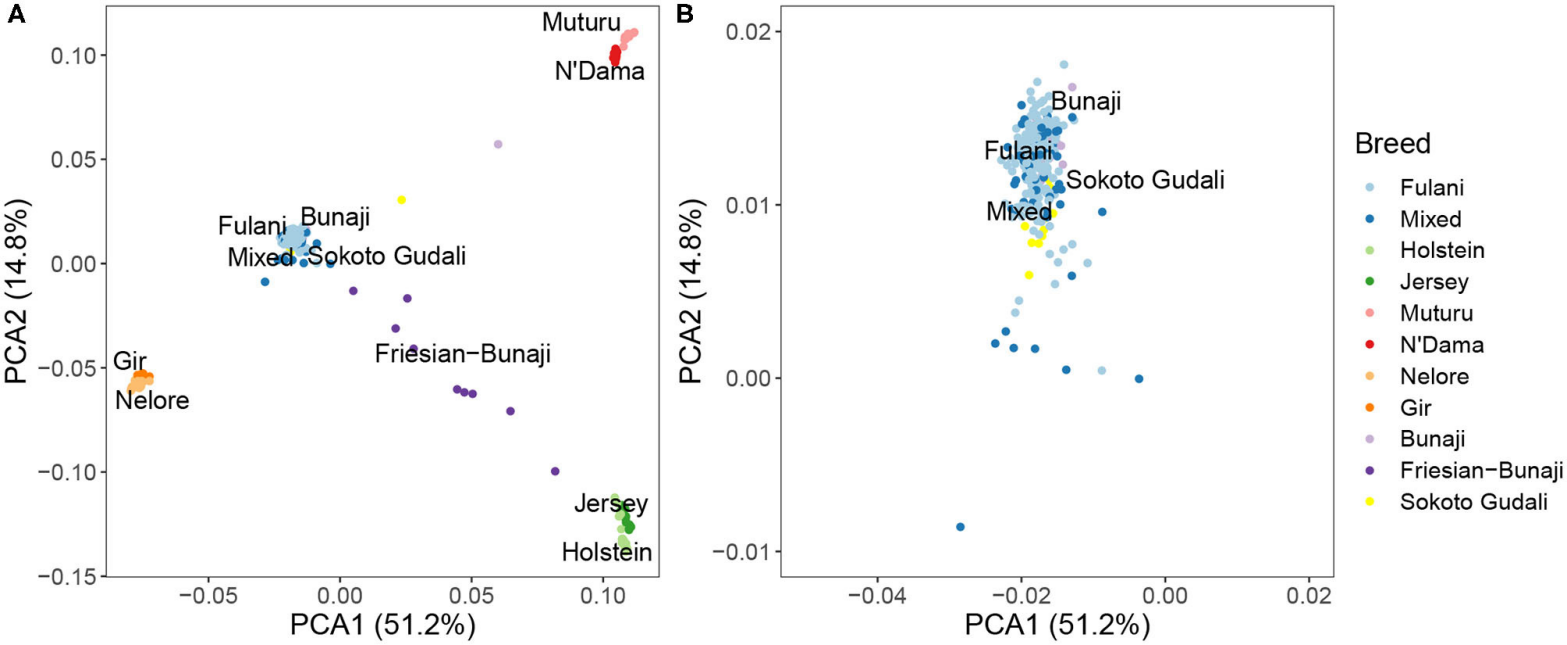
**(A)** PCA plot for the Fulani and mixed breeds with the reference cattle breeds for the first two principal components. **(B)** Zoomed in PCA plot showing the Fulani and mixed breeds.

PCA and hierarchical clustering analysis were used to show the population structure of the cattle breeds. Within the Cameroon cattle population, the PCA showed that the Fulani and mixed breed cattle cluster closely together along with the admixed Bunaji and Sokoto Gudali cattle (Figure 2). The African taurine, European taurine breeds, and Asian zebu breeds form their own distinct clusters. The first two components of the PCA account for 51.2 and 14.8% of the total variation, respectively.

**Figure 3:**
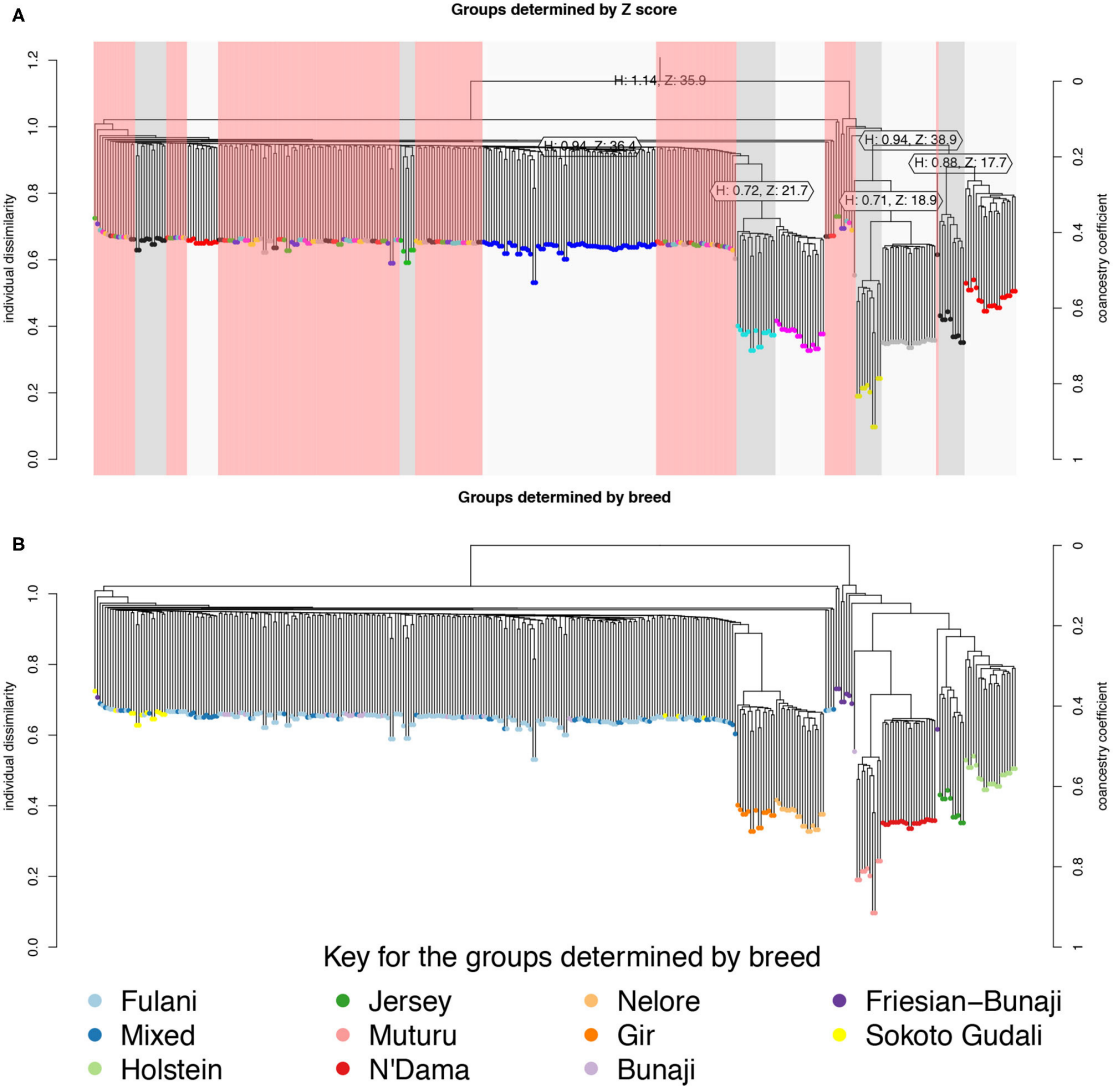
Hierarchical clustering of the IBS matrix. **(A)** Shows the groups as determined by individual dissimilarity and z-score, the color of the nodes represent membership to each of the clusters using a z-threshold of 15. The gray and white highlighted areas symbolize the cluster groups, each labeled with their unique cluster name. The red highlighted areas represent the outliers, which are made up of clusters with <5 individuals in them. **(B)** Shows the hierarchical relationship amongst individuals, the nodes are shaded according to the local abattoir employee's definition of breed.

The hierarchical clustering identified 10 large clusters of animals, shown by the gray and white bands and similarly colored nodes in Figure 3A. The remaining clusters were outliers, consisting of <5 animals in each group, and are highlighted by the red bands in Figure 3A. In Figure 3B, the same hierarchical relationship amongst individuals is shown as in Figure 3A, apart from the nodes are shaded according to the local abattoir employees definition of breed rather than cluster membership. In combination, Figures 3A,B confirm the PCA that the mixed breed cattle are grouped in the same cluster as the Fulani cattle and the admixed Bunaji and Sokoto Gudali cattle.

**Figure 4:**
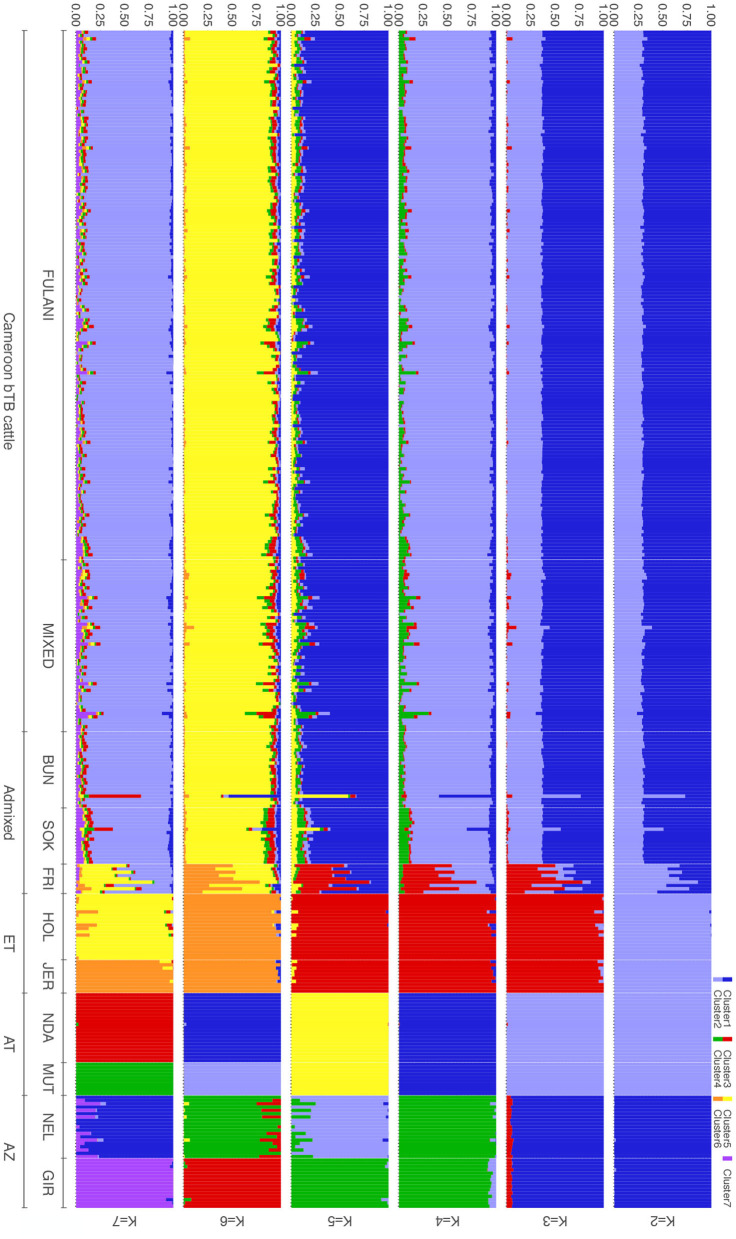
Admixture bar plots for the proportion of genetic membership to each ancestry assuming (*K* = 2 to *K* = 7) ancestral populations. Each animal is represented by a vertical line divided into K colors, indicating the likelihood of the animal's genome belonging to an ancestral population.

The population structure was investigated further using ADMIXTURE, with *K* = 2 to *K* = 7 shown in Figure 4. At *K* = 2 the cattle are split into the indicus and taurine groups. At *K* = 3, the African taurine and European taurine cattle diverge. The Fulani and mixed cattle have similar levels of African taurine and Asian zebu ancestries at the *K* = 3 level. The same pattern is observed in the supervised version of the admixture analysis (Supplementary Figure 1). In *K* = 4 to *K* = 7, the admixed cattle diverge, and higher levels of genetic heterogeneity in the admixed breeds compared to the indicus and taurine breeds is observed (Figure 4). Inspection of the cross-validation plot (Supplementary Figure 2) suggests that *K* = 7 is the optimal cluster number to describe the ancestry in this population. At *K* = 7, the Fulani and mixed cattle show similar levels of heterogeneity with some cattle having a larger proportion of indicus admixture then others (Figure 4). There is very little European taurine introgression in either the Fulani or mixed cattle.

#### 3.2.2. Genetic Differentiation, Inbreeding Coefficients, and Linkage Disequilibrium

High levels of genetic differentiation were observed between all the different breeds (Fst = 0.238; Mean Fst = 0.228; Range Fst = −0.058–0.871), however focusing on just the mixed and Fulani breeds there were low levels of genetic differentiation (Fst = 0.001; Mean Fst = 0.001; Range Fst = −0.009–0.14) indicating that there are high levels of shared genetic ancestry between the Fulani and mixed breed animals (Supplementary Figure 3).

**Figure 5:**
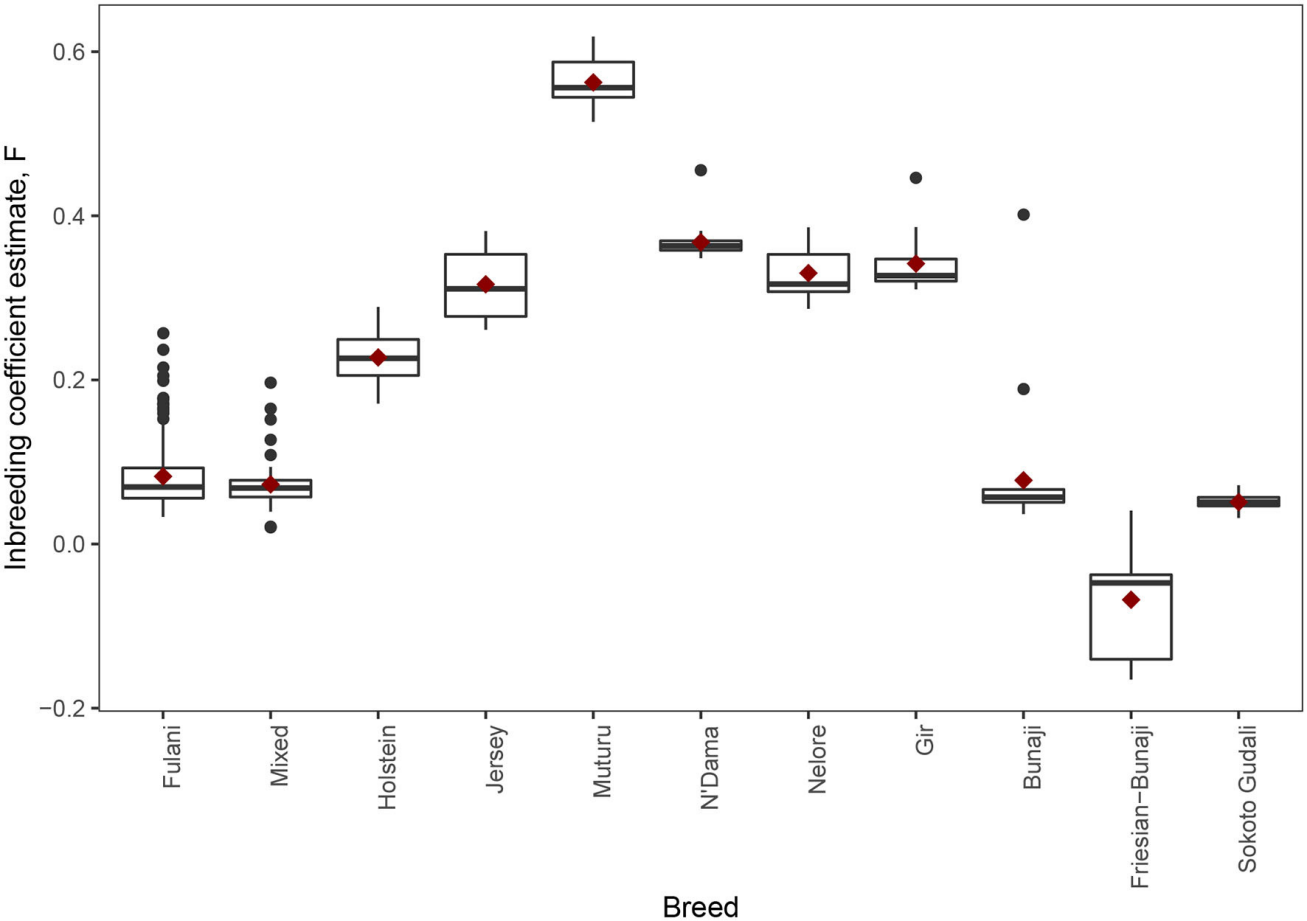
The observed inbreeding coefficient estimate, F, for each animal by breed. The red diamond represents the mean value for each breed.

The observed inbreeding coefficient, F is shown in Figure 5. Out of the animals we studied, the Muturu animals (which are an African taurine breed) have the highest level of inbreeding (mean F = 0.563, SE = 0.011) whilst the admixed Friesian-Bunaji animals were the most outbred group (mean F = −0.068, SE = 0.024). The Fulani cattle had a mean inbreeding coefficient of 0.082 (SE = 0.003) in comparison to 0.073 for the mixed cattle (SE = 0.004).

#### 3.2.3. Ascertainment Bias of the SNP Array

**Figure 6:**
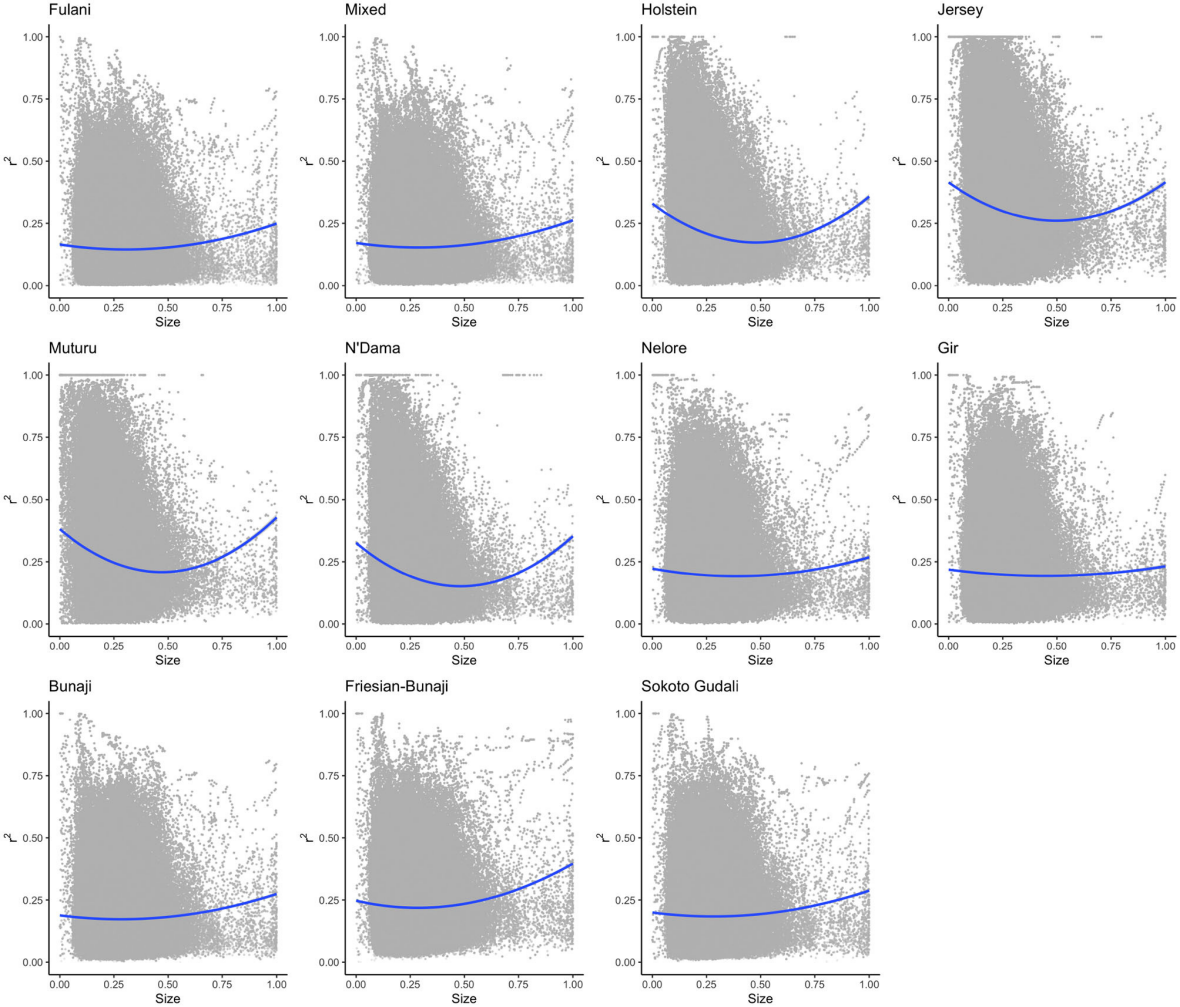
The linkage disequilibrium, *r*^2^ between pairs of SNPs in a 50 SNP window against the window's size (length in base pairs). For visualization purposes the x-axis (50SNP window size) was center scaled between 0 and 1. A second degree polynomial fitted to show the trend in *r*^2^ for each breed. The window size, representing the captured haplotype lengths and their average *r*^2^ (averaged across the SNPs within the window) shows the ascertainment bias of the array and its spacing design against the non-taurine breeds. Patterns of loss in LD and the range of LD differs between lineages based on the window size (affected by both genotypes and spacing of the them within the array).

**Figure 7:**
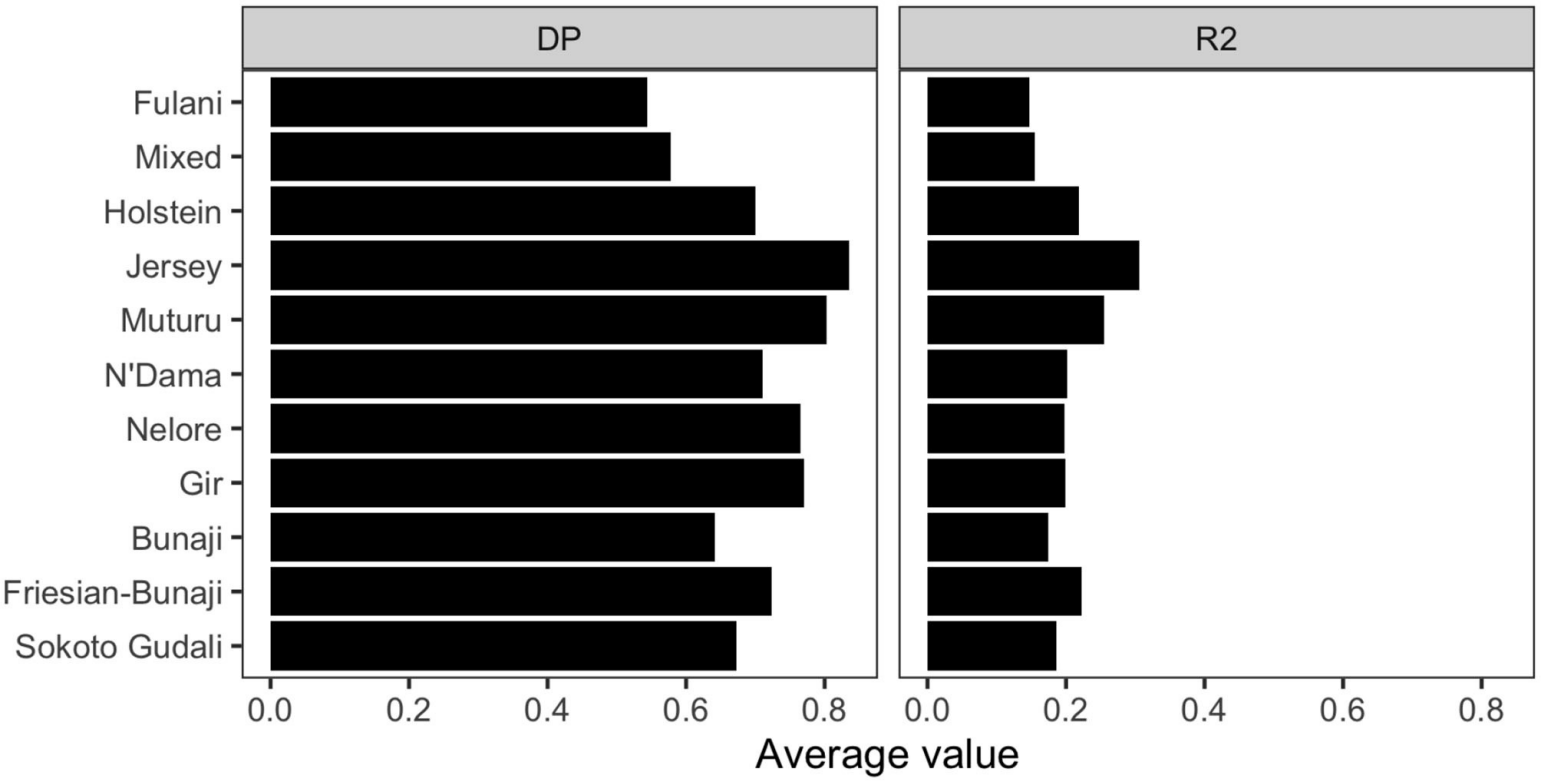
The average linkage disequilibrium as given by D′ and *r*^2^ for each breed.

The linkage disequilibrium, *r*^2^ between pairs of SNPs in a 50 SNP window against window size is shown in Figure 6. It shows the *r*^2^ follows a different trend for each of the breeds. Whilst there is a high level of ascertainment of SNPs from the European and African taurine breeds, the ascertainment of SNPs was a lot lower from the Asian (*Bos indicus*) zebu and admixed breeds (Figure 7). In particular, the SNP array does not have a good coverage of the Fulani or mixed breed haplotypes (Figure 6).

### 3.3. bTB Resistance Genetics

**Figure 8:**
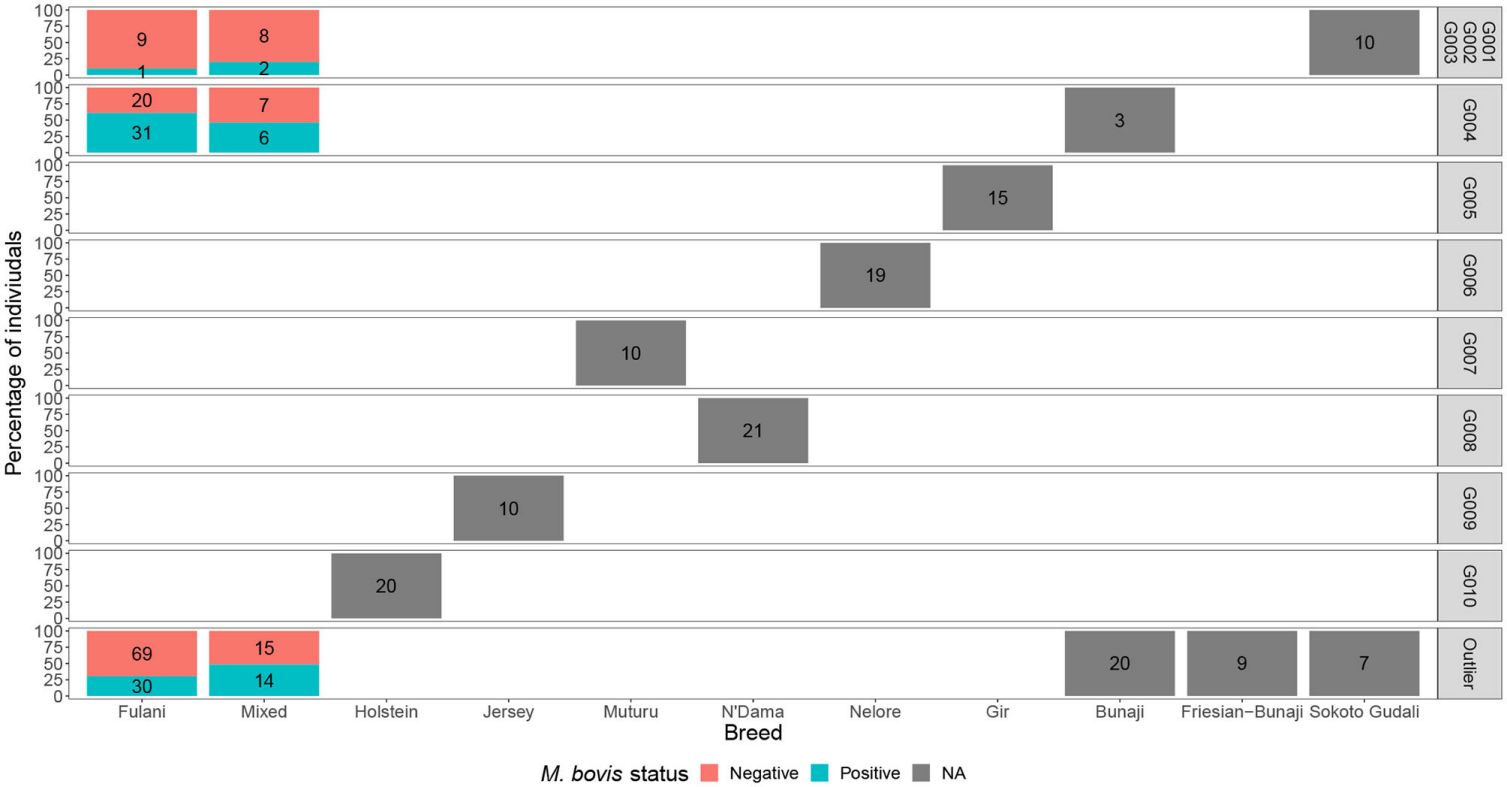
The percentage of individuals belonging to each hierarchical cluster group as shown in Figure 3 broken down by the local abattoir employees definition of breed and *M. bovis* status. The labels refer to number of individuals belonging to each group.

Although Kelly et al. (2018) observed that there was an association between bTB lesions and breed as defined by the local abattoir employees. Our model which looked at the association between *M. bovis* status and the local abattoir employees definition of breed, showed that there was no association with breed in the genotyped population (Table 1). However, when membership to hierarchical clustering group is used instead, there was a difference in risk of *M. bovis* between groups (Figure 8). Cattle belonging to cluster G004 were more than twice as likely to be *M. bovis* positive compared to cattle in the “outlier” cluster after accounting for age and sex (OR = 2.74, 95% CI = 1.38–5.54, Table 1) whilst there was no difference in risk for cattle in cluster G001/G002/G003 and the outlier cattle (OR = 0.39, 95% CI = 0.08–1.03, Table 1).

**Table 1:** Association between *M. bovis* and (a) local abattoir employees definition of breed and (b) hierarchical clustering group definition of breed after accounting for age and sex (*n* = 207).

**Predictors**	**Total number of individuals**	**Number *M. bovis* positive individuals**	**(a) Local abattoir employees definition of breed**			**(b) Hierarchical clustering group definition of breed**		
			**Odds Ratios**	**95% CI**	***p*-Value**	**Odds Ratios**	**95% CI**	***p*-Value**
Fulani	161	62	1.00					
Mixed	52	22	0.96	0.47–1.93	0.909			
Cluster Outlier	128	44				1.00		
Cluster G001/G002/G003	20	3				0.39	0.08–1.38	0.178
Cluster G004	64	37				2.74	1.38–5.54	0.004
Old	169	77	1.00			1.00		
Young	42	6	0.39	0.13–1.01	0.063	0.41	0.14–1.12	0.095
Female	139	73	1.00			1.00		
Male	69	7	0.13	0.05–0.29	<0.001	0.13	0.05–0.30	<0.001

**Figure 9:**
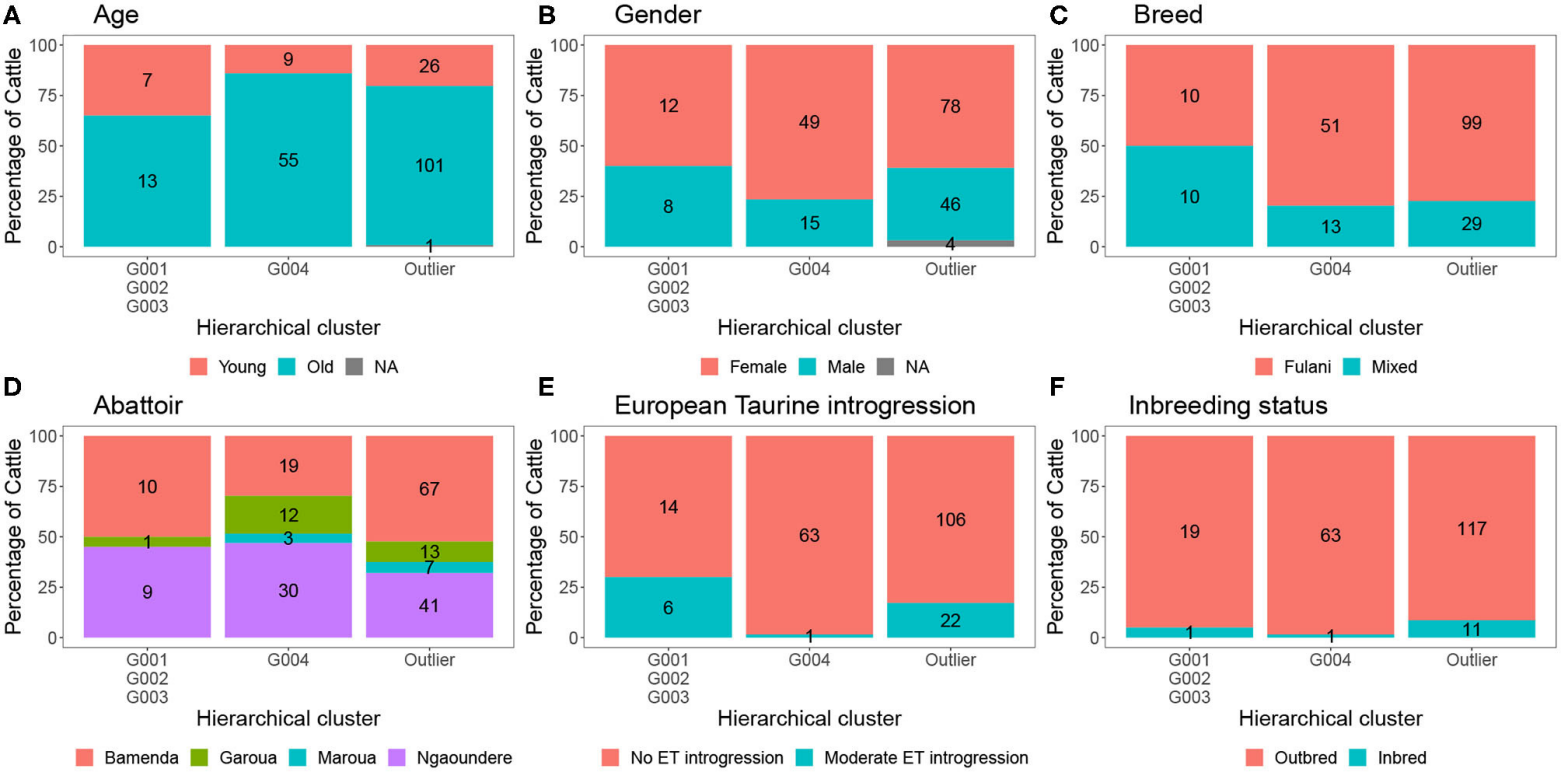
The number and percentage of animals in each hierarchical cluster according to **(A)** age, **(B)** gender, **(C)** local abattoir employees definition of breed, **(D)** abattoir, **(E)** European taurine introgression, and **(F)** inbreeding status. The labels refer to number of individuals belong to each group.

The characteristics of each cluster for the Fulani and mixed animals are described in Figure 9. There was no difference between cattle in G004 and the outliers in terms of the local abattoir employees definition of breed or inbreeding status. However, cattle in the outlier cluster were more likely to be ET introgressed then the G004 cattle (Fisher test, *p* <0.001). Yet, in a separate generalized linear models after accounting for age and sex there was no association between European taurine introgression and *M. bovis* (Table 2). In contrast, a further generalized linear model showed that inbred cattle have 0.08 times the odds of having *M. bovis* than outbred cattle, this means that inbred cattle were significantly less likely to be *M.bovis* positive than outbred cattle (OR = 0.08, 95% CI = 0.00–0.46, *p* = 0.014, Table 2).

**Table 2:** Association between (a) inbreeding and (b) European taurine introgression and *M. bovis* after accounting for age and sex (*n* = 207).

**Predictors**	**Total number of individuals**	**Number *M. bovis* positive individuals**	**(a) Inbreeding**			**(b) European taurine introgression**		
			**Odds Ratios**	**95% CI**	***p*-Value**	**Odds Ratios**	**95% CI**	***p*-Value**
Outbred	199	83	1.00					
Inbred	13	1	0.08	0.00–0.46	0.020			
No ET introgression	183	78				1.00		
Moderate ET introgression	29	6				0.39	0.13–1.03	0.068
Old	169	77	1.00			1.00		
Young	42	6	0.37	0.13–0.98	0.055	0.43	0.14–1.12	0.097
Female	139	73	1.00			1.00		
Male	69	7	0.12	0.05–0.27	<0.001	0.12	0.05–0.28	<0.001

#### 3.3.1. Heritability Analysis and Genome Wide Association Study

**Figure 10:**
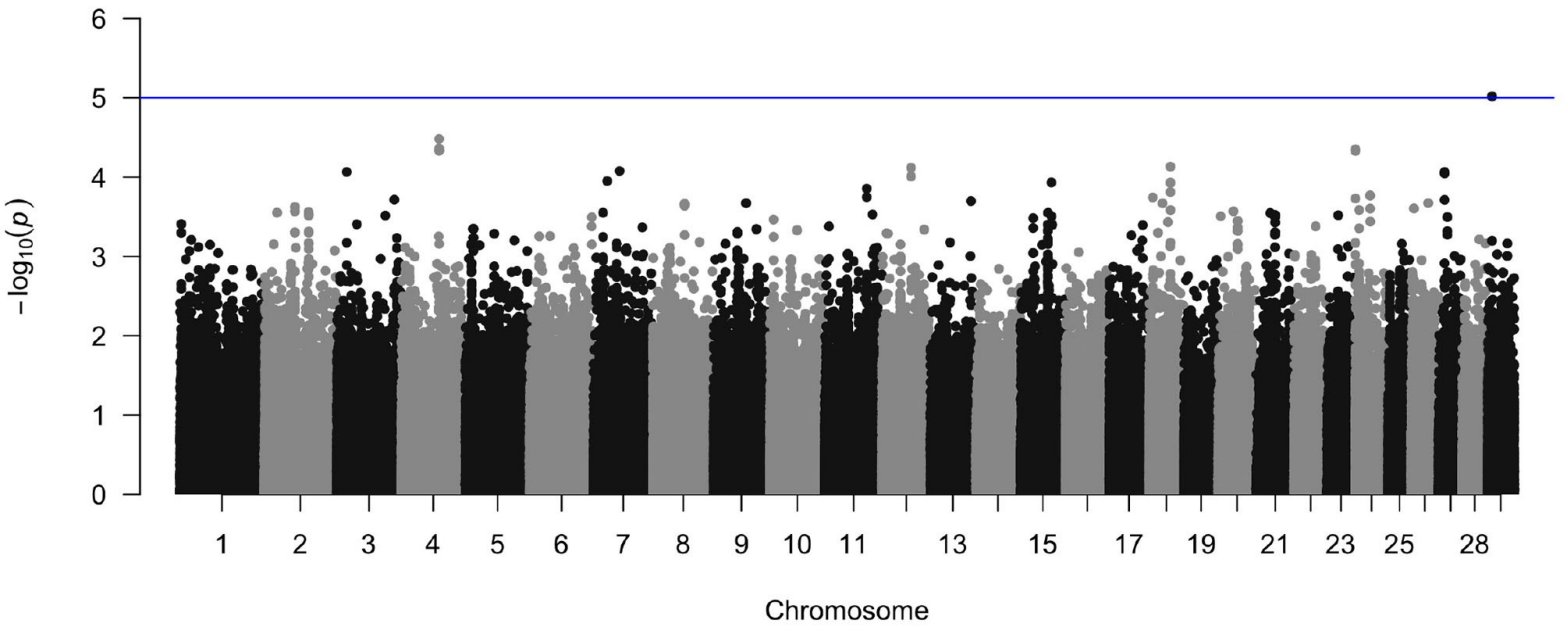
Manhattan plot of the genome wide association between SNPs and *M. bovis* status after accounting for age, sex, breed, and the first three principal components as covariates. The blue line represents the suggestive significance line of log(p) < 1 × 10^−5^.

The crude heritability of being infected with *M. bovis* in the Cameroon population, after accounting for age, sex, and breed is *h*^2^ = 21.7% (SE = 15.8, Supplementary Table 2). After accounting for age, sex, and breed, and the first three principal components there is a suggestion of an association between a SNP at Chr 29:9432267 and *M. bovis*, which passed the suggestive threshold rather than the genome wide threshold (*P* < 1 × 10^−5^, Figure 10 and Supplementary Figure 4 for the QQ-plot). This SNP is in a region previously highlighted as being possibly associated with bTB resistance in cattle by Richardson et al. (2016). Ensembl release 97 (Zerbino et al., 2017) identified one gene within ±100 Kbp of this region (29:9332267–9532267): phosphatidylinositol binding clathrin assembly protein (PICALM), which is involved in AP2-dependent clathrin-mediated endocytosis (Breuer et al., 2013).

## 4. Discussion

This study was aimed to investigate if there was a genetic basis to the relationship previously observed between the phenotypic definition of breed and bTB (Kelly et al., 2018). Although the local abattoir employees could identify a difference between the breeds, the apparent phenotypic differences in visual appearance between the breeds were not reflected by clear genomic differences. Within the Cameroon cattle population, the PCA showed that the Fulani and mixed breed cattle cluster closely together and the hierarchical clustering also grouped them together. Furthermore, the Fst analysis quantified the level of genetic differentiation between the two as very low (Fst = 0.001, Mean Fst = 0.001; Range Fst = −0.009–0.14) implying that the Fulani and mixed cattle are not genetically distinct. They also had similar levels of heterogeneity in the admixture analysis.

A study in East Africa has also compared farmer and field staff assessments of breed, based on phenotypic appearance with the admixture determinations of breed composition. They showed that phenotype-based assessments were very poor predictors of actual breed composition (*R*^2^ = 0.16) (Marshall et al., 2019).

Reasons for this discrepancy could be because breed may be a proxy for husbandry. Breed names can relate to the location where animals are found or ethnic group they are kept by rather than genetic differences between breeds (Frantz, 1993; Burnham, 1996; Rege and Tawah, 1999). Husbandry factors, such as pooled management (keeping all animals together and using bulls of the same breed) have also contributed to the low levels of genetic differentiation found between Fulani cattle (Ibeagha-Awemu and Erhardt, 2006).

Alternatively, as reflected from our linkage disequilibrium analysis, the marker diversity of the SNP chip does not accurately reflect the genetic diversity of the African admixed or *Bos indicus* breeds. Although the SNP array was validated in economically important beef and dairy cattle which encompassed both *Bos taurus* and *Bos indicus* breeds (Illumina, 2015), it did not include Fulani cattle in this process. This omission may have led to a lack of the discriminatory power which has been reflected in our results, meaning that it was not possible to differentiate between Fulani and mixed breed cattle in this study. The focus on dairy and beef breeds may mean that it is also missing variants responsible for disease resistance as well as observed physical characteristics. In the future, this analysis should be repeated with a custom SNP chip designed specifically for *Bos indicus* cattle to rule this out. These are currently being developed by the Centre of Tropical Livestock Genetics and Health (CTLGH) who are currently genotyping hundreds of cattle to generate a new SNP chip for African cattle populations which prioritizes SNPs for key traits, such as bTB resistance.

There was very little European taurine introgression from Holstein and Jersey breeds in both the Fulani and mixed cattle. Unlike other African countries, there has been very limited introduction of European breeds into Cameroon (Muwonge et al., 2019). Fulani cattle are kept by pastoral communities for dual purpose (meat and milk) and this lack of differentiation means that such a breed was less likely to be targeted for breed improvements by cross breeding with European taurine breeds. In addition, transhumance, the seasonal movement of livestock between pastures during the dry and wet season, practised by pastoralists in this region is dominated by native zebu breeds (Motta et al., 2018). Transhumance requires hardy, resilient and disease tolerant animals that can trek hundreds of miles along the Sahel transhumance highway (Motta et al., 2018; Turner and Schlecht, 2019). This limits the chances of breeding outside this gene pool since the characteristics targeted by breed improvements in most African settings are likely incompatible with the transhumance way of life.

Recently there have been introductions of exotic breeds and other taurine breeds into dairy improvement programs in Cameroon (Tambi, 1991; Njwe et al., 2002). These recent introductions will not be picked up in our sample of abattoir cattle as dairy cattle in Cameroon are very restricted in numbers and managed by small holders, who infrequently slaughter cattle or trade cattle with pastoralist groups (Kelly et al., 2016).

The literature contains many reports calling for the introduction of exotic breeds to be carefully managed. If uncontrolled cross-breeding continues without fully examining the socio-ecological, economical and environmental impact there is a risk of eroding the unique genetic resource which is adapted to the sub-Saharan environment (Ibeagha-Awemu and Erhardt, 2006; Mwai et al., 2015). The Fulani breeds are not only adapted for the transhumance way of life and the harsh climate (Hansen, 2004), it is known that other African cattle breeds have unique evolutionary adaptations to endemic diseases. For example, N'dama cattle are tolerant to trypanosomosis and East African Shorthorn Zebu from endemic areas of Kenya are tolerant to East Coast fever (Roberts and Gray, 1973; Coetzer and Tustin, 2004; Brown et al., 2005).

We showed that cattle in hierarchical clustering group G004 were more likely to be *M. bovis* positive than cattle in the “outlier” cluster. There was no difference in prevalence of *M. bovis* between individuals from G001/G002/G003 and the “outlier” cattle. Individuals in cluster G004 could be found across the whole study area, they were not restricted to one abattoir and they were equally likely to be Fulani or mixed breed. Cattle in the outlier cluster were however more likely to be moderately ET introgressed then the “outlier” cattle. Although preliminary and based upon small sample sizes, this result suggests that this attribute is ubiquitous in Cameroonian Fulani breeds and ought to be avoided to improve bTB control in such settings.

Furthermore, we found that inbred cattle were less likely to be infected with *M. bovis* than outbred cattle. The reasons behind this are not so clear. It is likely that small numbers could be driving the relationship since there was only one *M. bovis* positive inbred animal. However, with more data it may be worth investigating further. Many studies, including studies of cattle, show the opposite effect, that inbreeding depression has negative effects on host fitness (Coltman et al., 1999; Murray et al., 2013; Leroy, 2014). Alternatively, it is possible that the inbred cattle, are less likely to be cross-bred with exotic breeds so therefore they are less likely to become infected with bTB.

Kelly et al. (2018) showed that there was increased risk of having bTB-like lesions in Fulani cattle compared to the mixed breed group using the local abattoir employee's definition of breed in their study. Whereas, we showed that in our genotyped subset of this population, there was no association between local abattoir employee's definition of breed and *M. bovis*. There were a number of differences between the two analyses; the first was we only used a subset of individuals from the original Kelly *et al*. 2018 study. Secondly, we used individuals which where confirmed to have *M. bovis* by the Hain GenoType® rather than presence of bTB lesions to determine bTB status, so our case definition is narrower. To unpick this relationship, all the cattle would need to be genotyped which is beyond the logistical scope of this current study but merits further investigation when resources allow.

We found no association between European taurine introgression and *M. bovis*. However, Murray et al. (2013) found that Kenyan cattle with higher levels of European taurine introgression experienced more clinical illness. The difference observed here could be due to the substantially lower levels of introgression observed in Cameroon cattle (29 animals with 1.56–12.5% ET introgression), compared to the 113 animals with 2^−6^–36.1% ET introgression in Murray et al. (2013). Yet, the literature suggests that African zebu cattle are more resistant to bTB than exotic breeds of cattle (Ameni et al., 2007; Vordermeier et al., 2012).

In a cross-sectional study of bTB in Ethiopia, exotic cattle were more likely to be bTB positive using the comparative cervical intradermal tuberculin test (CIDT) than local cattle breeds (Habitu et al., 2019). A meta-analysis of bTB in Ethiopia also found that Holstein-Friesians have a higher bTB prevalence than local zebu [prevalence = 21.6% (95% CI: 14.7–30.7); prevalence = 4.1% (95% CI: 3.4–4.9), respectively (Sibhat et al., 2017)]. Furthermore, Ameni et al. (2007) found that Holsteins had more severe bTB pathology than zebu cattle in Ethiopia. Therefore, the use of and/or crossbreeding with taurine dairy cattle and the intensification of farming is likely to increase the incidence of bTB (Habitu et al., 2019).

Lastly, we investigated the role of additive genetic effects and individual SNPs on bTB resistance. We found that the heritability of bTB after accounting for age, sex, and breed in this study was *h*^2^ = 21.7% (SE = 15.8). The small sample size used for this heritability calculation has resulted in the large standard error.

Due to the polygenic nature of bTB resistance, there are few loci of large effect which could partially explains the lack of SNPs associated with *M. bovis* positive samples. In European taurine cattle there is strong evidence for genetic variation in resistance to bTB (Woolliams et al., 2008; Brotherstone et al., 2010; Bermingham et al., 2014; Tsairidou et al., 2014, 2018) with heritabilities ranging from 4 to 37%, depending on the phenotype used (Finlay et al., 2012; Bermingham et al., 2014; Richardson et al., 2016; Raphaka et al., 2017; Wilkinson et al., 2017; Tsairidou et al., 2018; Ring et al., 2019). This has led to the publication of genetic evaluations for resistance to bTB to allow farmers to select breeding sires with greater genetic bTB resistance (Banos et al., 2017). Importantly, these studies have also revealed that bTB resistance is polygenic (Woolliams et al., 2008; Bermingham et al., 2009, 2014; Brotherstone et al., 2010; Tsairidou et al., 2014, 2018).

After accounting for age, sex, breed, and population structure, our GWAS results showed that there was no evidence of a genetic association at the genome-wide significance level. At the suggestive level, there was an association between *M. bovis* and a SNP at Chr 29:9432267 (genome build *Bos taurus* UMD3.1). This SNP is in a region previously highlighted as being possibly associated with bTB resistance in cattle (Richardson et al., 2016).

Although our analyses suggest that bTB resistance is partly controlled by cattle genetics, it is possible that the heritability estimate and SNP effect are inflated due to the lack of knowledge of other potential confounders that are usually accounted for in genetic models. For example, we did not include population structure, polygenic additive effects or other systematic environmental effects which may confound the genetic effects. Furthermore, abattoir was excluded from these models due to the small sample sizes in each group. Breed and abattoir may be strongly confounded as the distribution of breeds across abattoirs is not uniform. So leaving out “abattoir” from the models has also increased the risk of falsely assigning breed or “cluster” effects to the additive genetic component of this model. More animals are needed to be genotyped before abattoir level effects can be included.

To conclude, there is a need to reconcile the differentiation between breed phenotype and genotypes of African cattle. We have shown that there is a lack of genetic difference between the apparent reported breeds. In addition, there is an indication of genetic variation in the resistance to bTB however this needs more evidence. Furthermore, we have highlighted the need for better tools to genotype African cattle populations.

Finally, when applying these findings to creating new selective breeding approaches it is important to understand the challenges faced by livestock in specific settings both in terms of pathogens and the environment, in addition to their intended purpose and how they fit into a defined management system. It is only at this point livestock keepers can then make informed breeding choices, not only against resistance to disease but breeding for production traits they require. By doing this it will create a more practical sustainable breed, which is adapted to different circumstances that fit in with the cultural context and local need. Without considering these wider potential impacts, breed improvement strategies could risk the unintended increase in incidence of diseases, such as bTB or adverse effects on production traits, thus massively affecting livelihoods.

## Data Availability Statement

The datasets presented in this study can be found in online repositories. The names of the repository/repositories and accession number(s) can be found below: https://doi.org/10.7488/ds/2722, University of Edinburgh DataShare Repository.

## Ethics Statement

The animal study was reviewed and approved by University of Edinburgh Ethical Review Committee.

## Author Contributions

BB, RK, AM, FE, and AD-W conceived and designed the study. RK, FE, LB, SM, and BB performed the field work. RC analyzed the data. AM, MS, LB, EC, and AD-W contributed the expertise and analysis tools. RC, AM, and MS wrote the first draft paper. All authors read and contributed to the final draft of the paper.

## Conflict of Interest

The authors declare that the research was conducted in the absence of any commercial or financial relationships that could be construed as a potential conflict of interest.
