# Supplementary material for: Genetic Diversity of Cameroon Cattle and a Putative Genomic Map for Resistance to Bovine Tuberculosis
Source: Front Genet. 2020 Nov 17;11:550215. doi: 10.3389/fgene.2020.550215 (PMC7705233; doi:10.3389/fgene.2020.550215)
Supplement: Supplementary file 1 [file Data_Sheet_1.PDF]

Supplementary Material

1 SUPPLEMENTARY DATA

The datasets supporting the conclusions of this article are available on the Edinburgh DataShare Repository. `https://doi.org/10.7488/ds/2722`

2 SUPPLEMENTARY TABLES AND FIGURES

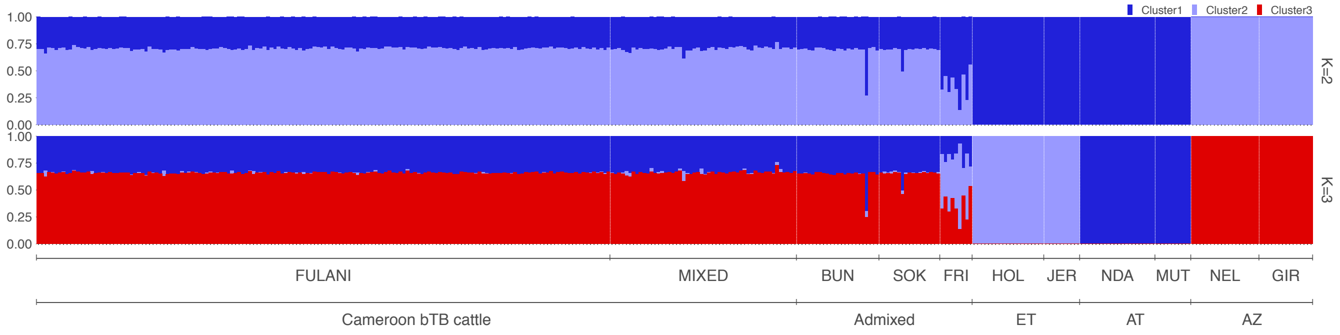

Figure S1. Admixture bar plots for the proportion of genetic membership to each ancestry assuming (K=2 to K=3) ancestral populations. The admixture analysis was run in the supervised, with European Taurine, African Taurine and Asian zebu (*Bos indicus*) and Admixed groups prespecified. Each animal is represented by a vertical line divided into K colours, indicating the likelihood of the animals genome belonging to an ancestral population

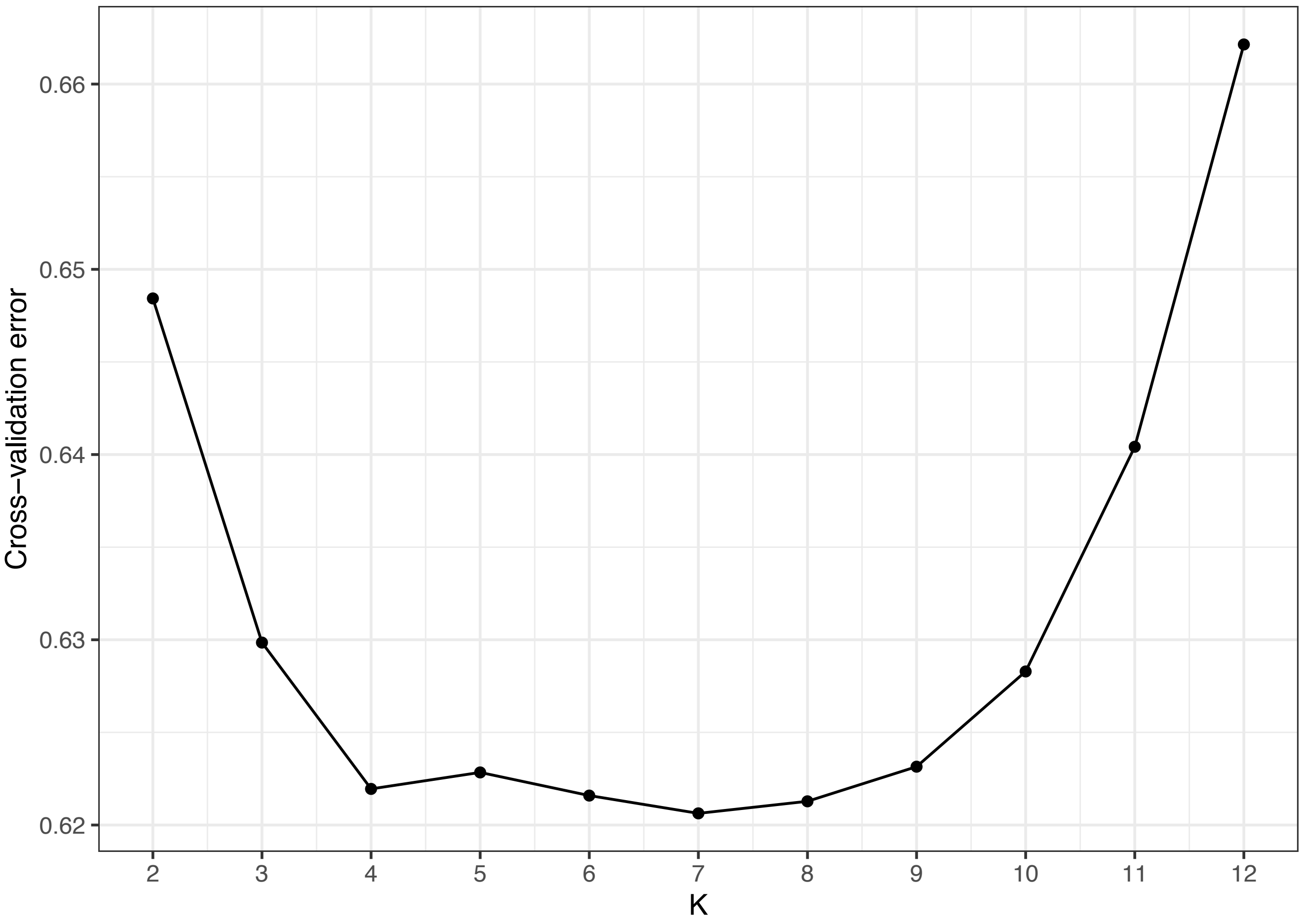

Figure S2. Cross-validation plot for the admixture analysis

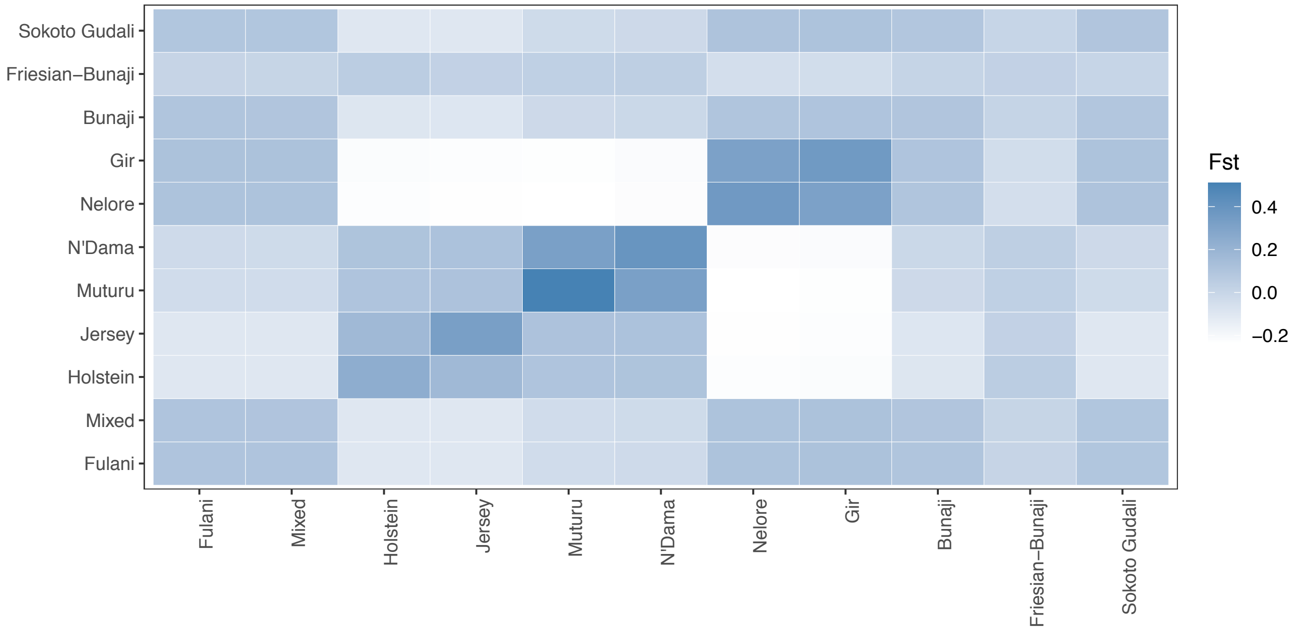

Figure S3. Relative beta estimator of Fst using Weir *et al.* 2002 Weir and Hill (2002) method comparing the Fulani and mixed breeds with the reference cattle

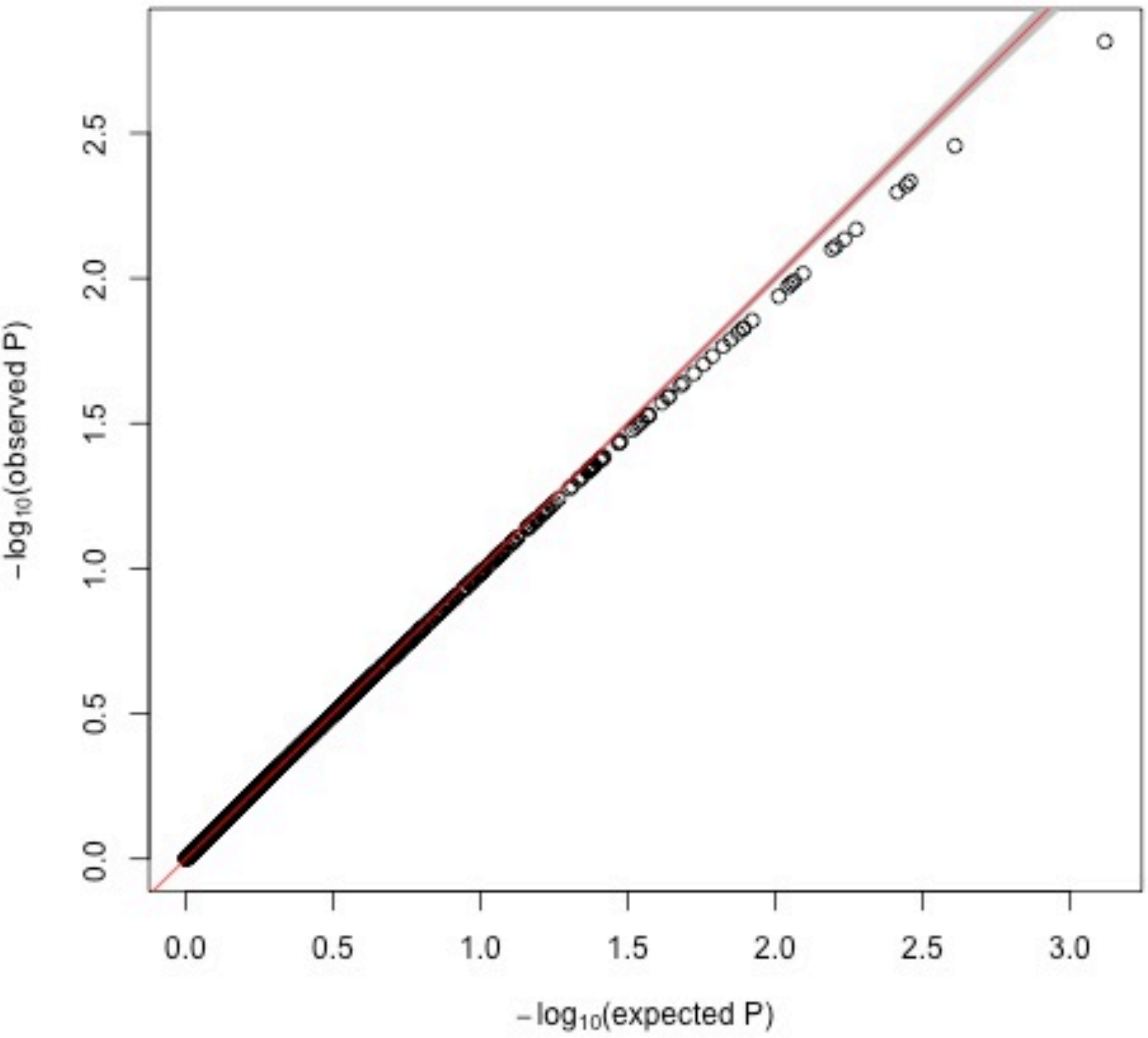

Figure S4. QQ-plot for the genome wide association between SNPs and *M. bovis* status after accounting for age, sex, breed and the first three principal components as covariates. Lamda = 1.003

Table S1. The studied cattle populations with number of genotyped individuals in the dataset before (N_{Before}) and after (N_{After}) quality control. Populations are defined according to DARGIS 2007 DAGRIS (2007).

Breed	Population	N_{Before}	N_{After}	Source
Fulani	Admixed	171	160	Kelly et al. (2018)
Mixed	Admixed	56	52	Kelly et al. (2018)
Holstein	European Taurine	63	20	Bovine HapMap Consortium (2009)
Jersey	European Taurine	36	10	Bovine HapMap Consortium (2009)
Muturu	African Taurine	10	10	Bahbahani et al. (2017)
N'Dama	African Taurine	24	21	Bahbahani et al. (2017)
Nelore	Asian Zebu	35	19	Bahbahani et al. (2017)
Gir	Asian Zebu	30	15	Bovine HapMap Consortium (2009)
Bunaji	Admixed	23	23	Bahbahani et al. (2017)
Friesian-Bunaji	Admixed	24	9	Bahbahani et al. (2017)
Sokoto Gudali	Admixed	21	17	Bahbahani et al. (2017)

Table S2. The heritability of *M. bovis* infection status. N = number of individuals; Npos = number of positive individuals *M. bovis*; Prevalence = prevalence followed by 95% confidence interval in brackets; VP = phenotypic variance; h^2 = heritability (%). Standard errors are in brackets

Response Variable	Fixed effects	N	Npos	Prevalence (95% CI)	VP (SE)	h^2 (SE)
M. bovis	None	212	84	39.6 (33.0 - 46.5)	4.31 (0.71)	23.37 (12.61)
M. bovis	Age and sex	207	79	38.2 (31.5 - 45.1)	4.16 (0.84)	20.75 (16.06)
M. bovis	Age, sex and breed	207	79	38.2 (31.5 - 45.1)	4.22 (0.85)	21.72 (15.84)
M. bovis	Age, sex, breed and abattoir	207	79	38.2 (31.5 - 45.1)	3.79 (1.60)	13.02 (36.62)

REFERENCES

Bahbahani, H., Tijjani, A., Mukasa, C., Wragg, D., Almathen, F., Nash, O., et al. (2017). Signatures of selection for environmental adaptation and zebu × taurine hybrid fitness in east african shorthorn zebu. *Frontiers in genetics* 8, 68

Bovine HapMap Consortium, A. (2009). Genome-wide survey of snp variation uncovers the genetic structure of cattle breeds. *Science* 324, 528–532

DAGRIS (2007). *Domestic Animal Genetic Resources Information System (DAGRIS)*

Kelly, R. F., Callaby, R., Egbe, N. F., Williams, D. J. L., Victor, N. N., Tanya, V. N., et al. (2018). Association of *Fasciola gigantica* co-infection with bovine tuberculosis infection and diagnosis in a naturally infected cattle population in africa. *Frontiers in Veterinary Science* 5, 214. doi:10.3389/fvets.2018.00214

Weir, B. S. and Hill, W. G. (2002). Estimating f-statistics. *Annual Review of Genetics* 36, 721–750. doi:10.1146/annurev.genet.36.050802.093940. PMID: 12359738
